# Supplementary figures and images for: Complete pan-plastome sequences enable high resolution phylogenetic classification of sugar beet and closely related crop wild relatives
Source: BMC Genomics. 2022 Feb 10;23:113. doi: 10.1186/s12864-022-08336-8 (PMC8830136; doi:10.1186/s12864-022-08336-8)

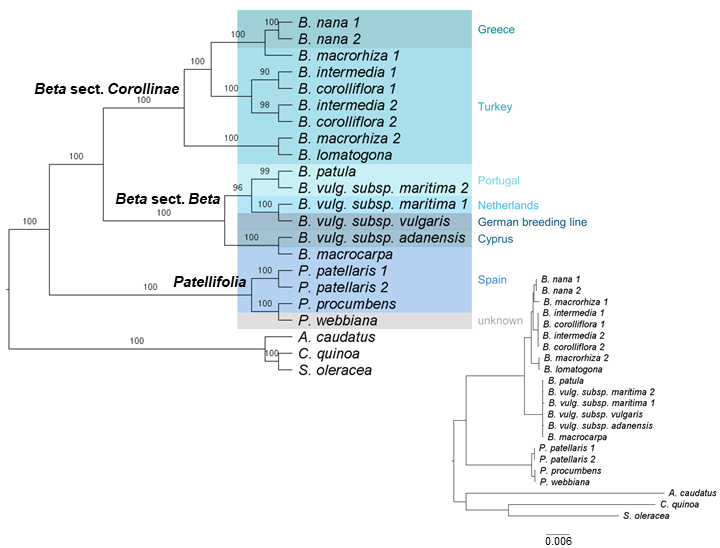

Supplement: Supplementary file 1 — Additional file 1: S1A.﻿ Geographic distribution of the Betoideae species as described in the literature. S1B. Distribution of coverage values and assembly length (in bp) for each region and the total assemblies. S1C. Circular and linear plots of selected plastome assembly sequences. S1D. Distance metrics for the comparison of splitstree results. S1E. Reduced phylogenetic tree based on 53 gene and intergenic regions. S1F. Phylogenetic trees based on different sequence matrices. S1G. Workflow for the construction of phylogenetic trees. [file 12864_2022_8336_MOESM1_ESM.pdf]
